# Dynamic differential evolution schemes of WRKY transcription factors in domesticated and wild rice

**DOI:** 10.1038/s41598-021-94109-4

**Published:** 2021-07-21

**Authors:** Anne J. Villacastin, Keeley S. Adams, Rin Boonjue, Paul J. Rushton, Mira Han, Jeffery Q. Shen

**Affiliations:** School of Life Sciences, University of Nevada Las Vegas, 4505 Maryland Parkway, Las Vegas, NV 89154 USA

**Keywords:** Evolution, Plant sciences

## Abstract

WRKY transcription factors play key roles in stress responses, growth, and development. We previously reported on the evolution of WRKYs from unicellular green algae to land plants. To address recent evolution events, we studied three domesticated and eight wild species in the genus *Oryza*, an ideal model due to its long history of domestication, economic importance, and central role as a model system. We have identified prevalence of Group III WRKYs despite differences in breeding of cultivated and wild species. Same groups of WRKY genes tend to cluster together, suggesting recent, multiple duplication events. Duplications followed by divergence may result in neofunctionalizations of co-expressed WRKY genes that finely tune the regulation of target genes in a same metabolic or response pathway. WRKY genes have undergone recent rearrangements to form novel genes. Group Ib WRKYs, unique to AA genome type *Oryza* species, are derived from Group III genes dated back to 6.76 million years ago. Gene tree reconciliation analysis with the species tree revealed details of duplication and loss events in the 11 genomes. Selection analysis on single copy orthologs reveals the highly conserved nature of the WRKY domain and clusters of fast evolving sites under strong positive selection pressure. Also, the numbers of single copy orthologs under positive or negative selection almost evenly split. Our results provide valuable insights into the preservation and diversification of an important gene family under strong selective pressure for biotechnological improvements of the world’s most valued food crop.

## Introduction

Transcription factors are key players in the well-orchestrated transcription regulation of all organisms. In plants, one of the most prominent gene families encodes WRKY transcription factors^1^. They play key roles in plant immune responses^2,3^, responses to abiotic stresses such as salt and drought^4^, and hormones such as abscisic acid^5^.

WRKY genes are only present in green plants, fungi, Amoebozoa, and fornicate^6,7^. Their high specificity to and rapid expansions in plants hint at their key roles in evolution from single cellular aquatic algae, gradually developing varied defense strategies to combat biotic and abiotic stress agents, and ultimately becoming multicellular flowering organisms. Hence, studies of the WRKY gene family can provide understandings into these evolution processes. Recent data support lateral gene transfer of plant WRKY genes to non-plant organisms^6^. WRKYs found in algae have unique characteristics that are not homologous in sequence to flowering plants, further backing the theory that their presence could be due to lateral transfer that occurred millions of years ago^6^.

Current knowledge on WRKY genes does not provide the evolution of the WRKY family within a particular genus, or more importantly, between closely related species. A primary goal in evolutionary biology studies is to understand the mechanisms underlying the diversity of species. Genetic diversity, gene duplication, divergence, or gene loss events can lead to speciation. Large changes in gene family size are believed to highly impact evolution that leads to fixation of complex traits and adaptive phenotypes that may determine the fitness of the species^8–11^. Closely examining related lineages, e.g., in a genus, helps address gene family evolution within the genus. This can provide insights that may be more valuable than genomic comparison studies among different genera.

Rice variety has been greatly increased within a relatively narrow evolutionary time frame, approximately 15 million years. Within this intense domestication process and limited time scale, several speciation events have occurred^12^. Domesticated Asian rice (*Oryza sativa)*, which is the most widely exploited member of the genus, has 21 wild relatives within the genus^13^ and a domesticated relative endemic in Africa (*Oryza glaberrima*). To date, eleven of these genomes have been fully sequenced including the two popular subspecies, *O. sativa* subsp. *japonica* and *indica.* In this study, the genomes of the following species within the genus *Oryza* were thoroughly screened for the identification of putative WRKY genes: *O. barthii* (African wild rice, genome type AA), *O. glaberrima* (African rice, genome type AA)*, O. brachyantha* (grass rice, genome type FF), *O. glumaepatula* (Brazilian wild rice, genome type AA), *O. meridionalis* (Australian wild rice, genome type AA), *O. nivara* (Indian wild rice, genome type AA), *O. punctata* (red rice, genome type BB) and *O. rufipogon* (brownbeard rice/Asian wild rice, genome type AA). An appreciable amount of genetic resources is well maintained within the genomes of these wild variants that can be employed for further development of cultivated rice. The present study provides invaluable insights into the molecular evolution of an imperative family of transcription factors to aid in the understanding of the diversification of duplicated genes or gene losses under strong selective pressure. It also contributes to the pool of useful information for the biotechnological improvement of a staple food crop.

## Results and discussion

### The WRKY superfamily dynamically expanded in *Oryza*

**Figure 1:**
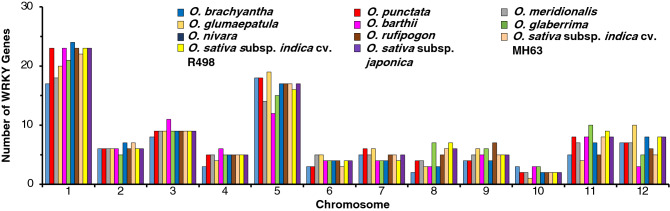
WRKY gene distributions are consistent across *Oryza*. On average, chromosome 1 contains the greatest number of WRKY genes (24%), chromosome 5 contains 17% of total identified WRKY genes and chromosome 10 contains the least number of WRKY genes (only 2%). Bar heights represent number of WRKY genes distributed across the *Oryza* genomes, bars are clustered per chromosome, colors indicate *Oryza* species genes are identified in. Ordered from left to right roughly based on lineage.

**Figure 2:**
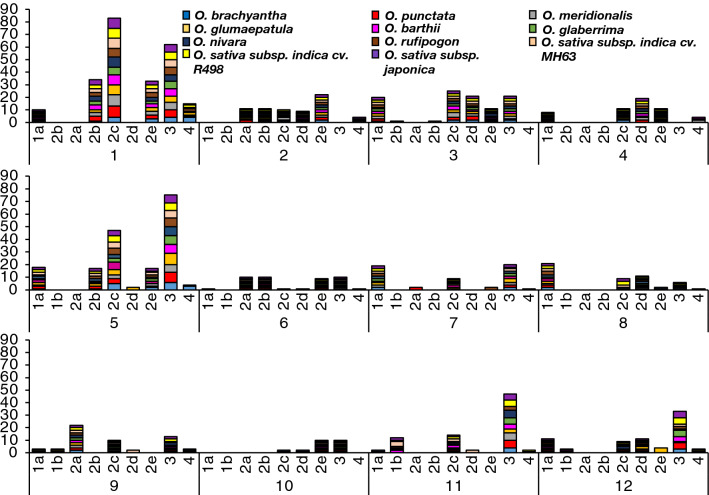
WRKY groups are consistent across *Oryza* but are not equally found across chromosomes. Stacked columns represent the number of WRKY genes identified under the WRKY group classification for each of the 12 chromosomes in *Oryza*. Different colors represent different *Oryza* species. Species are arranged based on divergence from the *Oryza* ancestor, from oldest to youngest.

Analysis of the 11 Oryza genomes reveal a total of 1,018 WRKY genes (Table 1 and Supplementary Table S1). Genes are distributed across the 12 chromosomes of these diploid Oryza species, with chromosome 1 having the greatest number of WRKYs (24%), followed by chromosome 5 (17%) and chromosome 3 (10%; Fig. 1). Chromosome 10 is the least populated by these genes with only 2% of all the WRKY genes identified. WRKY genes are categorized based on the classification criteria documented in the Methods and Materials section. Group III is the largest in all species, with an average of 28 genes/species, followed by Group IIc with an average of 21 genes/species. A majority of these Group III and IIc types are localized on chromosomes 1 and 5 (Fig. 2, Supplementary Fig. S1). Chromosome 1 is the longest of the 12 *Oryza* chromosomes, ranging from ~ 33.9 to ~ 46.5 Mb, while chromosome 5 ranges from ~ 20.1 to ~ 31.2 Mb across all 11 genomes.

**Table 1:** Classification of WRKY genes within the *Oryza* genus.

*Oryza* species		Group classification									Total WRKY	Genome type	Estimated genome size (Mb)	Sequenced genome SiZe (Mb)	Ave. number of WRKY per Mb
		Ia	Ib	IIa	IIb	IIc	IId	IIe	III	IV					
Wild	*O. barthii*	9	3	3	7	23	7	10	28	5	95	AA	411	308	0.31
	*O. brachyantha*	9	0	3	4	19	4	11	24	9	83	FF	362	261	0.32
	*O. glumaepatula*	10	2	4	7	19	7	11	28	5	93	AA	464	373	0.25
	*O. meridionalis*	10	1	4	6	20	7	10	27	3	88	AA	435	336	0.26
	*O. nivara*	11	2	4	7	22	7	11	28	2	94	AA	448	338	0.28
	*O. punctata*	11	0	5	8	20	8	10	30	2	94	BB	423	394	0.24
	*O. rufipogon*	12	3	4	7	20	7	11	27	3	94	AA	445	338	0.28
Cultivated	*O. sativa* subsp. *japonica* cv. Nipponbare	11	2	4	8	21	7	11	31	3	98	AA	387	373	0.26
	*O. sativa subsp. indica* cv. MH63	11	4	4	7	24	7	10	25	2	94	AA	386	360	0.26
	*O. sativa indica* cv. R498	11	1	4	7	25	7	10	30	3	98	AA	391	390	0.25
	*O. glaberrima*	10	1	4	6	16	7	10	31	2	87	AA	358	316	0.28

Group II and III WRKYs are only found in the green plant lineage and have continued to expand during plant evolution^14,15^. This is also true for the *Oryza* genomes in this study. Members of this WRKY group were believed to be more advanced^16^, due to more diverged C_2_HC zinc finger motifs and high adaptability success. Our results are consistent with the report that the number of Group III WRKYs is markedly higher in monocots than in eudicots^17^. Several rice Group III genes are involved in pathogen resistance^18^, drought and cold tolerance^19^. It is possible that this lineage specific expansion of Group III WRKY genes is associated with the continued development of inducible defense mechanisms in monocots.

### Some WRKY genes with shared functions are clustered together on chromosomes

Supplementary Fig. S2 shows the graphical representation of all the identified WRKY proteins as they are distributed on all 12 chromosomes of the 11 *Oryza* genomes used in this assay. In general, WRKY proteins that are within orthologous groups and the same WRKY classification groups are found in similar regions of the chromosomes across the *Oryza* genomes. Examples include chromosomes 1 and 5, and the short arm of 11 and 12. The presence of homogenous type WRKY clusters is a prevalent organizational theme for this gene superfamily within land plants^20^, suggestive of recent, multiple duplication events. This distinct clustering of WRKY genes may be a consequence of function, that is, WRKY genes that are more concentrated within a chromosome may be involved in co-regulating genes that play roles in the same metabolic pathway or response. In an exhaustive transcriptional study of Asian rice under abiotic stress and phytohormone treatments^21^, several tandemly duplicated WRKY genes located in chromosomal clusters were shown to be co-expressed. For example, two Group IIa genes in *Oryza sativa* ssp. *japonica, OsjaWRKY62* and *-76*, are located only 19 kb apart on chromosome 9 (Supplementary Fig. S2). Both transcripts were shown to be highly expressed in mature leaves and young roots and treatment of growth phytohormone auxin or gibberellic acid up-regulated the expression of both genes in two-week old seedlings^21^. Even more compelling is the cluster of genes found on the chromosome 11 distal tip hot spot (Supplementary Fig. S2). All four Group III WRKYs, *OsjaWRKY40, -46a, -50* and *-100*, were highly expressed in mature panicles and up-regulated upon treatment of salicylic acid. This coordinated expression owing to physical location of genes is also apparent *in Ananas comosus* (pineapple), where a cluster of three WRKYs on chromosome 19, *AcWRKY46*, *-47*, and *-48*, were all highly expressed in the roots (fold change > 2) under cold stress^22^.

### A large number of WRKY genes resulted from tandem duplication

Rapid evolution has been linked to large multigene families, especially ones that are involved in disease resistance, reproduction, and morphological changes^12^. Studies of these multigene families support the birth-and-death model of gene evolution^23,24^. In this model, duplication drives the birth of new genes and some genes are kept in the genome for extended periods while others get lost or become nonfunctional through accumulated mutations. While cultivated Asian species contain most WRKY genes, 26 genes are lost in either one of the four domesticated species (Supplementary Table S2). Two of these genes are found only in the wild species. Nine of these WRKYs have acquired mutations during the *Oryza* expansion, when the gene either had modifications in its WRKY domain or lost a domain completely (detailed below).

Random mutations can also cause duplicated genes to diverge in sequence from their parental genes and in some cases acquire new functions. Overall, 14–29% of rice genes occur as tandem duplicates^25^. We have identified multiple cases of tandem duplication events that not only increased the copies of WRKY genes within the *Oryza* genomes, but also led to the formation of two domain WRKYs via the tandem duplication of a WRKY domain. This is especially evident in the Group Ib WRKYs. Group Ib only has 19 identified members in all 11 *Oryza* genomes, making it the least predominant type of WRKYs. They are all present on chromosomes 9, 11 and 12, except *OnivWRKY61*, which is located on chromosome 3 of *O. nivara* (Supplementary Fig. S2). These WRKY proteins have two WRKY domains with C_2_HC zinc finger motifs, unlike Group Ia WRKY genes, which have C_2_H_2_ zinc finger motifs.

**Figure 3:**
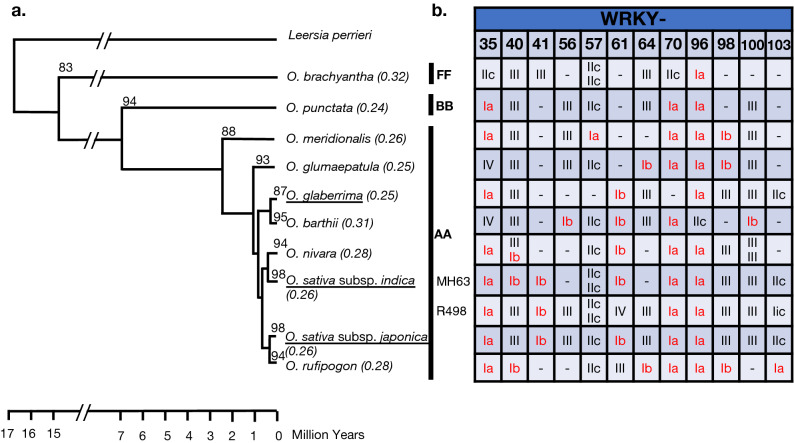
Evolution of the WRKY transcription factors and domain duplication events across *Oryza* genomes within the evolution timeline. (**a**) *Oryza* species phylogenetic tree with branches drawn to scale in evolutionary timeline^28^. The number of identified WRKYs is shown on each branch. The WRKY gene density (Average number of WRKYs/Mb of estimated genome size) is shown in parentheses, cultivated species identified in underlined font and genome types in bold font to the right. (**b**) WRKY genes with different domain structures across the *Oryza* genomes. Group classification is listed for each *Oryza* species. Classifications with two WRKY domains are in red font, double classification in a cell means gene occurs as paralog within the species.

**Figure 4:**
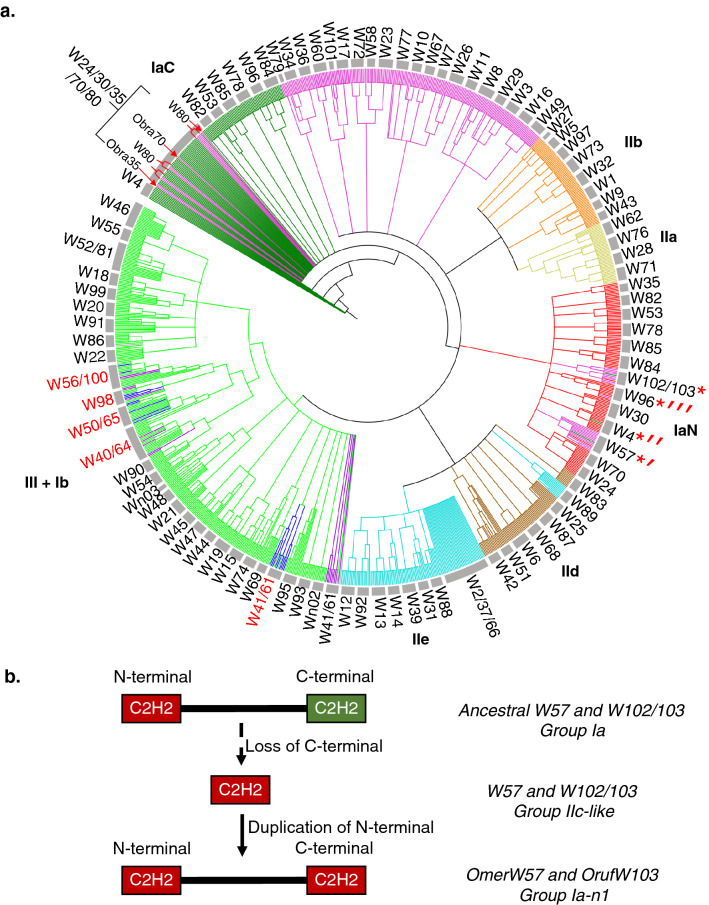
Phylogenetic construction of WRKY proteins from *Oryza* and hypothesized domain evolution. (**a**) Maximum likelihood tree of WRKY domain sequences using 1000 bootstrap replicates. Group IaN: red, Group IaC: dark green, Group IbN: blue, Group IbC: purple, Group IIa: yellow, Group IIb: orange, Group IIc: pink, Group IId: brown, Group IIe: cyan, Group III: light green, and Group IV: black. Clades have also been labeled according to orthologous group placement. Clades notated with two or more WRKY IDs indicate an intermixed clade. Red asterisks indicate novel group 1a WRKYs resulted from duplications of the N-terminal domain of more ancient group Ia proteins. Red arrows indicate novel WRKYs likely resulted from loss of the N-terminal WRKY domain of Group Ia proteins. Red font texts indicate clades with Group Ib. Nodes with bootstrap support < 50 are not shown. (**b**) Proposed hypothesis for how the clades observed in panel A for W57 and W102/103 could have occurred via the rapid loss and gain of WRKY domains. The hypothesis proposes that a Group Ia ancestor for each respective WRKY may have lost its C-terminus and left a IIc-like N-terminus, which subsequently duplicated within select lineages to create a novel Group Ia-like WRKYs (Group 1a-n1).

*WRKY98* is particularly interesting. It shows that even within the same genome type, with an estimated mean divergence of only 2.41 million years, we observe a rapid domain loss or duplication just within the same WRKY gene (Fig. 3). *WRKY98* is a Group III gene (with one WRKY domain) in *O. sativa* subsp. *japonica*, *O. sativa* subsp. *indica*, *O. glaberrima,* and *O. nivara*. However, *O. glumaepatula*, *O. meridionalis*, and *O. rufipogon* are Group Ib WRKY genes (with two WRKY domains). Both the domains from the Group Ib WRKY98 proteins are in a monophyletic clade with the domains of Group III WRKY98 proteins (Fig. 4). Our hypothesis for this intermixing is based on the estimated phylogeny that ranks *O. glumaepatula* and *O. meridionalis* as the oldest species that contain WRKY98. It is possible that the WRKY domain was lost after speciation from *O. glumaepatula*, and subsequently, *O. rufipogon* regained its second domain in a more recent event via tandem duplication. Furthermore, *O. glumaepatula*, *O. rufipogon* and *O. meridionalis* appear to have also formed via tandem duplication, since both domains fall within the WRKY98 clade. This work supports that gain and loss of WRKY domains has occurred multiple times during the evolution of plants, and evidence of recent events is common in *Oryza* species.

### A subset of Group Ib genes, specific to the AA genome of *Oryza*, resulted from fusions of two Group III genes

Further inspection of these Group Ib WRKY genes derived from Group III WRKYs sheds light on their formation. In some cases, the new gene is a fusion from two genes, as exemplified in the two pairs of paralogs, *WRKY40* with *WRKY64* and *WRKY50* with *WRKY65*. *WRKY40/64* are Group III genes in some species but Group Ib genes in others (Fig. 3). The N-terminal WRKY domains of these Group Ibs are in the Group III WRKY50/65 clade while their C-terminal WRKY domains fall into the Group III WRKY40/64 clade (Fig. 4). The clustering is more clearly illustrated by their protein sequence alignments (Supplementary Fig. S3), where the sequences show similarities that explain why certain domains clustered the way they did. Closer inspection of the transcript structure of these genes reveals the joining of a Group III gene (*WRKY40/64*) with another Group III gene (*WRKY50/65*) to form Group Ib WRKYs (Supplementary Fig. S4). Interestingly, these fusions were found for both sets of paralogs, *WRKY40* fused with *WRKY50* and *WRKY64* fused with *WRKY65*. They are located on two different chromosomes and appear to have happened independently across several species, and hence cannot be explained by pure coincidence.

These fusions create dual-WRKY-domain proteins with binding affinities derived from two genes. They may be able to bind to the target genes of both original, Group III transcription factors. *WRKY40/64* and *WRKY50/65* are present as single domain Group III genes in *O. sativa,* except for the *indica* cultivar MH63 where *WRKY40* is a Group Ib. These genes have been associated with increased expression upon rice fungal blast infection^26^*.* Another interesting feature is that the fusion gene has brought DNA binding from WRKY40/64 under the control of the *WRKY50/65* promoter. This may result in neofunctionalization but answering this question requires additional studies. Both sets of these fusion proteins were detected in *O. rufipogon*, cultivated Asian rice’s immediate wild progenitor and an excellent candidate species for crossbreeding with domesticated rice^27^.

Our data provide evidence for the timing of the formation of these Group Ib genes from two copies of Group III genes. These Group Ib genes were present specifically in the AA genome of *Oryza* but not *O. brachyantha* (FF genome type) and *O. punctata* (BB genome type) (Fig. 3), suggesting that they evolved in *Oryza* earlier than 6.76 million years ago when the AA genome type *Oryza* species diverged from the BB genome type^28^.

### Domain deletion followed by tandem duplication produced a novel group of Ia proteins in some species

Interestingly Group IIc WRKY proteins are present in the IaN and IaC clades (Fig. 4a). In part, this is due to the broad definition for Group IIc proteins, a single WRKY domain containing a CX_4_C zinc-finger. Similarly, the zinc-fingers of Group Ia WRKYs are also defined by the same CX_4_C sequence, but they have two WRKY domains. Typically, the broadness of definition and overlap between Ia and IIc is not a problem, as indicated by the fact that most Ia cluster with other Ia and most IIc cluster with other IIc (Fig. 4a). However, we present cases where there is conflict between the operational definitions due to unique circumstances.

WRKY102/103 in all of the studied genomes contain only one WRKY domain except for that in *O. rufipogon*, which has two WRKY domains. Based on the classification criteria, these single WRKY domain proteins belong to Group IIc. However, in Fig. 4a, WRKY102/103 WRKY domains are imbedded in the Group IaN clade instead of the Group IIc clades, indicating that the protein has a higher similarity to Group IaN domains. This suggests that Group IIc WRKY102/103 were derived from the N-terminal WRKY domain of Group Ia proteins, and that the C-terminal WRKY domain was lost. Interestingly, the two WRKY domains of OrufiWRKY103 (represented by a red and a green line next to the red asterisk, respectively, Fig. 4a) are also clustered in the IaN clade. Since the duplicated N-terminal domains are only present in *O. rufipogon*, we hypothesize that the remaining N-terminal WRKY domain of a Group Ia WRKY recently duplicated to produce a new type of Group Ia protein in *O. rufipogon*. WRKY57 (labeled with *′, Fig. 4a) also appears to have a similar story as WRKY102/103, serving as novel examples of domain evolution within the WRKY duplicated N-terminal WRKY domain of Ia (Fig. 4b).

There are examples where both loss and duplication has occurred to only a single species, demonstrating the rapid evolution of WRKY within *Oryza*. WRKY4 proteins are Group Ia WRKYs; however, both domains of OnivWRKY4 are clustered in the WRKY4 clade (labeled with *′′, in Fig. 4a), suggesting that OnivWRKY4 appears to have lost its C-terminal domain and rapidly experienced tandem duplication of its N-terminal domain, all within the *O. nivara* species. If it was not within a single species, we would have observed the process of loss and duplication occur in stages as observed with WRKY102/103. WRKY96 (also labeled with *′′′) experienced only domain loss, but not tandem duplication. Like *O. rufipogon*, *O. barthii* evolved much later in the genus, but it is the only species that lost the C-terminal domain of WRKY96 but has not duplicated its N-terminal domain.

Our data also revealed the opposite scenario—only the C-terminal WRKY domain is retained. WRKY35 and − 70 in *O. brachyantha* (pointed by red arrows in Fig. 4a) are clustered with Group IaC WRKY domains. They likely resulted from loss of a more ancient Group Ia N-terminal WRKY domain. WRKY80s are Group IIc WRKY proteins by classification; however, their domains are clustered with the IaC clade, suggesting that the genus lost the N-terminal domain prior to the evolution of these studied species. However, we have not identified examples for duplication of the C-terminal WRKY domain in the *Oryza* genomes we have analyzed so far.

Though the example of WRKY102/103 and the similar cases suggest that domain-loss and -duplication occur among the Group Ia WRKYs, there are very few existing cases despite the extensive age of the Group Ia WRKYs as a sub-class. This would suggest that domain loss and duplication within Group Ia is not very frequent or that these changes cause a loss of function and deteriorate.

### A large number of WRKY genes on chromosomes 11 and 12 evolved from two recent segmental duplication events

**Figure 5:**
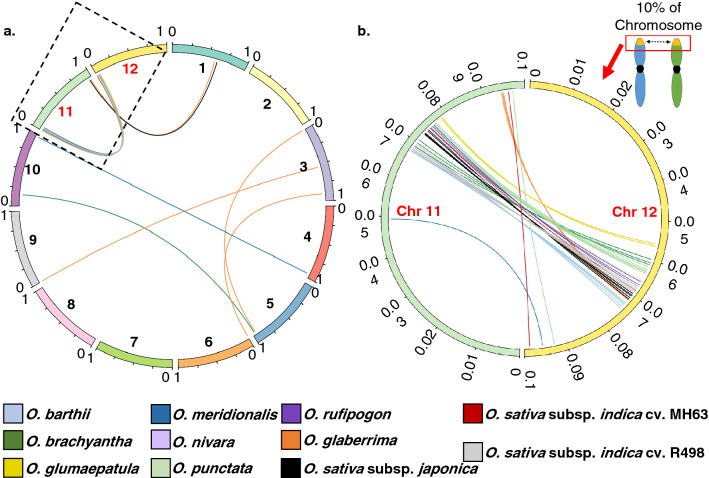
WRKY paralogs identified across the *Oryza* genomes. To compare chromosomes across different species, the chromosomes sizes were made to scale from 0 to 1. Paralogous relationships are represented by a line, indicating the position of a WRKY gene on each end. Each WRKY gene position is relative to its respective species on the 0 to 1 scale. (**a**) Syntenic relationship between 44 interchromosomal WRKY paralogs across all 12 chromosomes (**b**) Region enclosed in dashed box in A shows segmental duplication events at the distal 10% region of chromosomes 11 and 12 short arms have generated ~ 38% of total identified paralogs.

There are 66 sets of duplicates within species paralogs, 22 of which occur in tandem whereas 44 emerged through segmental duplications (Fig. 5a). Syntenic analysis of these interchromosomal paralogs shows that a majority are located between the distal short arms of chromosomes 11 and 12 (Fig. 5b). The number of pairs for these two chromosomes alone could be as low as 20% to as much as 100% of the total paralogs identified per species. As many as 32 pairs of WRKY paralogs were found on chromosomes 11 and 12, nearly half of the 66 total paralogs identified for all 11 *Oryza* genomes.

Analysis of duplicated genes not only reveals the principal origin of new genes within the genomic sequence, but also how functional divergence in genes occurs even when those genes are practically subjected to the same environmental constraints and/or selective pressures^29^. Duplication of chromosome 11 to 12 has been reported to date back as far as 21 MYA, far later than the estimated origin of the grass family ~ 55–70 MYA^30^. This duplication is then followed by what is considered as the most recent segmental duplication event in rice on the 3 MB short arm of both chromosomes, ~ 7.7 Mya^31^. This large-scale duplication certainly explains the burst of WRKY genes in especially the top 6% of the two chromosomes (Fig. 5b). The impact of the duplication event on WRKY genes and, more importantly, the consequence of having multiple copies of these genes within the plant genome warrants future studies.

### WRKYs evolved into a new WRKY group and even different gene families

The most common type of switch from one group to another within the WRKY gene family in *Oryza* is partial loss of WRKY domains, i.e., the WRKY or zinc finger motif, resulting in Group IV WRKYs^5^. High sequence similarities are found between these Group IV WRKYs with other orthologous WRKYs (Supplementary Table S3). Most of the Group IV WRKYs identified in this study retain the N-terminal WRKY motif more often than the C-terminal zinc-chelating motif.

To better understand the evolutionary significance of these sequence changes, we investigated changes of WRKY domain architecture. In the so-called “WRKY signature”, the majority of the WRKY proteins exhibit the WRKYGQK (866 occurrences) consensus sequence, followed by the WRKYGEK (72) and the less conventional WRKYGKK (51) (Supplementary Fig. S5). We have observed modifications of the motif where the conserved WRKY amino acid sequences are replaced by WKKY (11), WRMC (10), WSKY (3), and WVKY (3). WKKY and WRMC are only identified in Group IIc proteins, except the latter was found in a single Group Ia WRKY in *O. rufipogon* and has double copies of the WRMC motif. Both WSKY and WVKY are found in Group Ib and III proteins where the unique motif is found only on the N-terminal end of the double domain containing proteins. These sequence modifications of the highly conserved WRKY motif can be assumed to be newly evolved and its occurrence mostly in the N-terminal domain might be suggestive of the functional importance of conserving the C-terminal domain WRKY. The C-terminal WRKY domain has been identified to be mainly responsible for DNA binding^1^ whereas reports on the functional binding of the N-terminal domain are scarce. While there are far fewer instances of variants in the WRKY signature WRKYGQK amino acid sequence in dicot species^6^, rice has more variants in the WRKY signature sequence. Further work will establish whether this is monocot- or *Oryza*-specific.

WRKY TFs that contain more than two WRKY domains are exceedingly rare. ObraWRKY24 is categorized under Group Ia and has three copies of the WRKY motifs. The N-terminal WRKY contains the double WRKYGQK motif followed by the C_2_H_2_ zinc ion binding pocket, while the C-terminal WRKY domain contains the typical single WRKYGQK—C_2_H_2_ zinc finger architecture. This protein’s ortholog in *O. sativa* has been linked with response to fungal blast infection, jasmonate hormone regulation for disease resistance, and crosstalk of gibberellins and abscisic acid for germination^32^. *O. brachyantha*, native to Africa, is resistant to rice pathogens such as bacterial blight, yellow stemborer, leaf folder, and whorl maggot^33^. Further functional analysis of this WRKY may shed new light on its role in the wild species’ high resistance against biotic pathogens.

### Gene tree reconciliation analysis with the *Oryza* species tree revealed details of duplication and loss events in the 11 *Oryza* genomes

**Figure 6:**
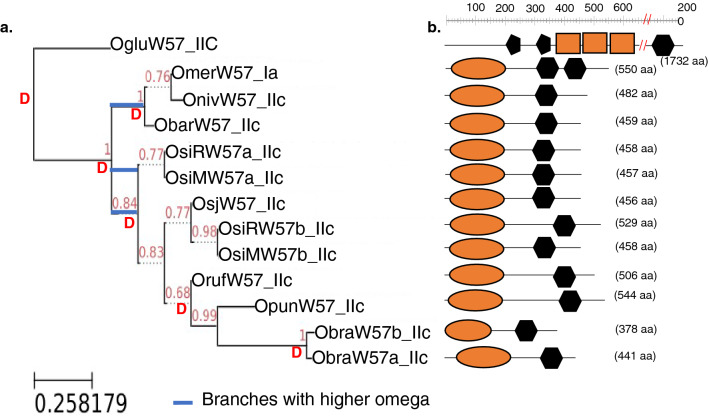
Estimation of gene duplication and loss events in the 11 *Oryza* species. (**a**) Reconciliation of rearranged maximum likelihood tree of OsW57 genes across *Oryza* genomes with the species tree (Fig. 3) using NOTUNG v2.9. (Duplication-Loss Model). D represents duplication events. Nodes indicate bootstrap values for 1000 replicates. Blue bar indicates branches with higher ω values. (**b**) Graphical view of ScanProsite predicted domain features are presented on the right. SPHERE: ULP protease; PENTAGON: EF-hand calcium-binding; HEXAGON: WRKY domain; RECTANGLE: solute carrier (SOLCAR).

To infer the domain evolution of WRKYs across the *Oryza* genomes, we performed a reconciliation of the *Oryza* species tree (Fig. 3a) with the rearranged maximum likelihood tree of representative *WRKY57* orthologs (Fig. 6a). This analysis reveals at least six duplication events for the *WRKY57* gene within the *Oryza* species used in the assay. The oldest *WRKY57* duplication event is suggested to have occurred before the divergence of *O. glumaepatula*. Three more duplication events occurred within the AA genome types, one that led to the ortholog in *O. punctata* (BB genome type) and the last distinct duplication event that led to the paralogs of *O. brachyantha* (FF genome type). The only other genomes with paralogs of *WRKY57* are the two *indica* subspecies of *O. sativa* which each had an extra copy of the gene.

ScanProsite analysis using full protein sequences of the WRKY57 across the *Oryza* genomes reveal the conserved domains, except for the highly diverged OgluW57 that lacks the ULP-protease and instead has two copies of the EF-hand calcium-binding domain profile and three of the Solute carrier (SOLCAR) repeat profile (Fig. 6b). This explains the divergent branching of *O. glumaepatula* from the AA genome branches. Ubiquitin-like proteins (ULP) are involved in the covalent modification of proteins linked with crucial cellular pathways such as apoptosis, the G2-M DNA damage checkpoint of the cell cycle and stress responses^34^. EF-hand calcium-binding domain profile and SOLCAR are both substrate binding proteins, with the former being specific for the universal secondary messenger Ca^2+^, suggesting a critical role in diverse signaling mechanisms. The fusion of different domains and generation of chimeric proteins can lead to a novel function different from the individual isoforms. Some reported altered functions of chimeric proteins comprise modified localization and tissue specificity which may be linked with diseases^35^. Presence of these important domains joined with the WRKY domain may suggest either the combined functionality of these domains with a transcription factor or dual functionality of the protein depending on localization and/or specificity. These new observations expand on our previous work that showed that flowering plants contain proteins with domains typical for both resistance (R) proteins and WRKY transcription factors^6^. R protein-WRKY genes have evolved numerous times in flowering plants, each type being restricted to specific flowering plant lineages. The *japonica* rice contains two such genes and *indica* one gene. These chimeric proteins contain not only novel combinations of protein domains but also novel combinations and numbers of WRKY domains. Once formed, R protein-WRKY genes may combine different components of signaling pathways that may either create new diversity in signaling.

In the tree, both the *OgluW57* branch and the most recent common ancestor of the two *ObraW57* are the longest branches of the tree. Although not shown in the domain diagram of Fig. 6b, the *ObraW57* do have divergent gene structures compared to the rest of the orthologs as seen in Supplementary Fig. S6.

To further inspect adaptive evolution after gene duplication, branch model analysis using the CODEML program in the PAML v4.0 suite was used to test whether branches after duplication events are evolving under different constraints. We found that of the 94 duplication events inferred across the 102 gene trees of different WRKY families, 18 duplication events led to significantly different ω ratios (Supplementary Fig. S7, Table S4). In all but one of those 18 duplications, the branch after duplication showed a higher ω compared to the rest of the tree indicating relaxed selection after duplication, with some of the ω’s significantly larger than 1. For *WRKY57*, for example, rate of evolution significantly increased after duplication node r31 [ω0 (0.54) < ω1 (1.01)], which is suggestive of a relaxation of selection constraints^36^ for the succeeding AA genome types of *Oryza*. Genes that experience faster rates of evolution have been hypothesized to result to phenotypic plasticity in response to changing environments^37^. Given WRKYs’ importance in a myriad of plant functions, this observation further supports the hypothesis that these genes are directed towards novel functions to adjust to environmental variations. Interestingly, only one gene (*WRKY71)* showed slower rate of evolution after a duplication event, which could indicate a less dispensable nature of the gene.

### Selection predominantly occurred outside of the WRKY domain and numbers of single copy orthologs under positive or negative selection almost evenly split

**Figure 7:**
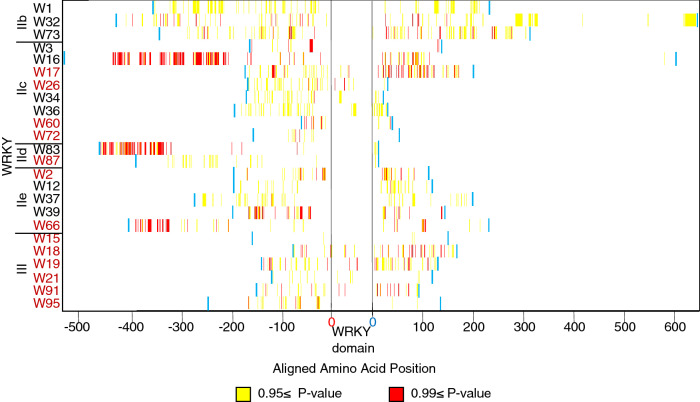
Analysis of sites under highly significant positive selection suggests high conservation of the WRKY domain throughout the *Oryza* genus with mutations predominantly occurring outside the domain. Representative single copy ortholog WRKYs are arranged according to group shown on the left. WRKYs in red font are determined to be highly conserved based on the site model PAML tests in Table 2 (Supplementary Table S7). Double domain containing Group I WRKYs were not included in the diagram. Amino acids are aligned relative to the location of the WRKY domain with red and blue 0 indicating first and last amino acid of the domain. Bayes-Empirical-Bayes estimates of dN/dS at each amino acid were analyzed using PAML; yellow bars represent sites with p-values ≥ 0.95 and red bars p-values ≥ 0.99. Non-significant data (p-value < 0.95) is not indicated. Blue bars mark the start and end of each WRKY protein.

Positive selection acting upon protein-coding genes is one of the main driving forces that result in the birth of new gene motifs. This could eventually lead to genes with novel functions after duplication events have occurred. Using the ‘site models’ in the PAML package, we deduced and determine codon sites undergoing positive selection within the sequence of orthologous WRKY proteins across the 11 *Oryza* species. As shown in Table 2 and Supplementary Table S5, based on the two models, the dN/dS ratios (ω) for 17 WRKY proteins are greater than 1.0, suggesting that ~ 49% of the 35 orthologous WRKY proteins were under positive selection. Typically, positive selection only acts on specific sites or regions of proteins in a small number of lineages in a phylogenetic tree. For this reason, we compared the different site models of the CODEML program to identify which regions of the WRKY proteins are under positive selection. Closer inspection of these 17 proteins indicates that amino acid selection diversity is largely insignificant in the WRKY domain (Fig. 7), different from the rest of the protein sites. This is especially true for Groups IIb, IId, and IIe, which essentially lack positive selection sites in the domains. The data support conclusions from functional studies. Disruption of the functional WRKY domain regions has been associated with loss of function of the protein and disturbance in downstream gene expression, often involved in stress response^38^. Structural analysis of the WRKY-DNA binding complex has previously shown that tryptophan, tyrosine and two lysine of the conserved WRKYGQK motif are vital for the protein’s DNA binding ability. In addition, the zinc finger motifs have also been shown to be essential for binding^39^.

**Table 2:** Maximum likelihood estimation (Likelihood scores for tests for selection among codons of WRKY using site models implemented in PAML is detailed in Supplementary Table S5. Parameter estimates under models of variable ω ratios among sites for Oryza WRKYs are detailed in Supplementary Table S9) of selective pressure shows 17 single copy WRKY orthologs evolving under positive selective pressure.

Group	17 positively selected WRKY genes	18 conserved WRKY genes (negative/neutral selection)
I	35, 85, 96	4, 24, 78
IIa	76	62
IIb	1, 32, 73	
IIc	3, 16, 34, 36	17, 26, 60, 67, 72
IId	83	87
IIe	12, 13, 37, 39	2, 66
III	45	15, 18, 19, 21, 91, 95

About 51% of the 35 orthologous WRKY proteins, most of which belong to Groups IIc and III, were under purifying or negative selection (ω < 1, Table 2). Among these, *OsjaWRKY24* (a Group Ia) has been shown to function as a negative regulator of both the gibberellic acid and abscisic acid signaling^40^. Overexpression of *OsjaWRKY62* (a Group IIa) impaired resistance of *Oryza sativa* against bacterial blight^18^. Hence, some negatively selected WRKY genes involved in defense remained essentially unaltered throughout the evolution of the *Oryza* genomes.

### Genus-specific analysis reveals novel WRKY groups specific for the AA genome type *Oryza* species

Our previous study^6^ investigated and described the evolution and origin of the WRKY gene family. We proposed two hypotheses by which the WRKY family may have expanded. In the “Group I Hypothesis,” all WRKY genes evolved from the C-terminal of the Group Ia WRKY genes. In the second hypothesis, “IIa + b Separate Hypothesis,” Groups IIa and b evolved from an early WRKY domain instead of diverging from Group Ia.

**Figure 8:**
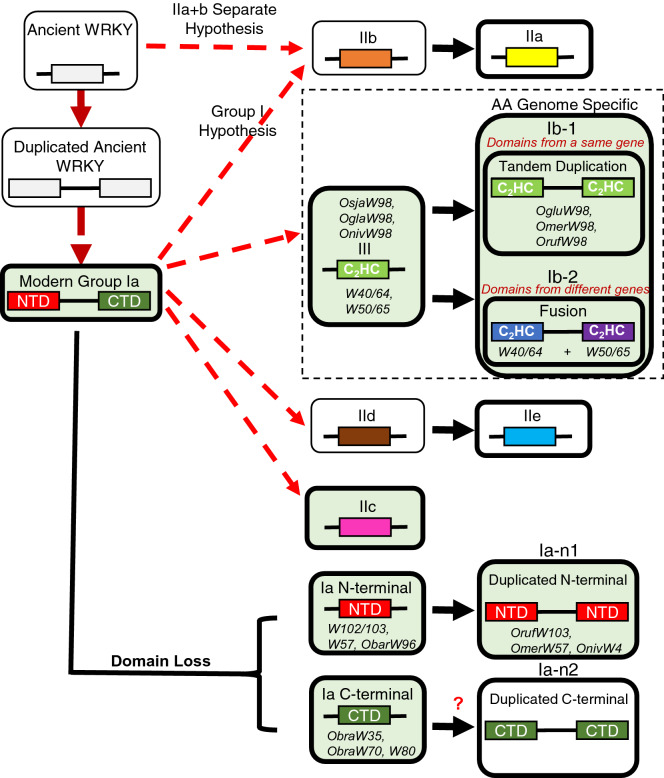
Evolution of the WRKY family through domain duplication and deletion. The figure summarizes how the WRKY family in *Oryza* is expanding and evolving according to the phylogenetic analysis. Red dashed arrows indicate relationships as we reported previously^6^. Green filled boxes represent new information deduced from our data with specific *Oryza* WRKY gene examples. WRKY domains in the black dashed rectangle are the C_2_H_2_ type while the rest are the C_2_HC type. Tandem duplication of the N-terminal WRKY domain of more ancient Group Ia proteins produces Group Ia-n1. The question mark indicates that the tandem duplication of the C-terminal WRKY domain of more ancient Group Ia to produce Group Ia-n2 is theoretically possible but has not been found in the *Oryza* genomes we have studied so far.

Conducting a phylogenetic analysis of all the identified WRKY proteins across the 11 *Oryza* species reveals much about the recent evolution of the WRKY gene family in *Oryza*. Given the close relationships among the species in this study and the evolutionary time frame of 15 million years^28^, we produced a phylogenetic analysis indicating high conservation of WRKY genes. As expected, most orthologous groups fell into their own monophyletic clades. However, the resolution and insight offered by a genus-specific analysis allowed to make conclusions that are not possible with a single species or multi-genus study. Figure 8 shows the proposed evolution scheme of WRKY genes in the *Oryza* genus. This study has shown a unique class of WRKYs that contain two copies of Group III WRKY domains. They are specific for AA genome type *Oryza* species, suggesting these emerged around 6.8 MYA after the split from the BB genome. These “Group Ib” genes are birthed through tandem duplication (Ib-1) or fusion (Ib-2) of non-homologous C_2_HC-zinc finger Group III WRKYs. The original classification of WRKY proteins into Groups I, II, and III was based partly on phylogeny and partly on the number of WRKY domains in the proteins^1^. Our results show the limitations of the original classification that puts proteins with two WRKY domains into Group Ia or Ib. Ib-2 WRKYs are distinct from Ib-1 WRKYs in structure and origin, but they fall under the same category in the original classification (Fig. 4a). We also identified a scheme to produce a novel two WRKY domain group (Group Ia-n1, Fig. 8). On the other hand, WRKY35 and − 70 are examples of a Group Ia WRKY losing its N-terminal WRKY domain. Although we do not have a specific example of the C-terminal WRKY domain being duplicated, as is the case for Group Ia’s N-terminal domain, this begs the question whether such an event can be observed in other genomes.

## Conclusion

Comparative genomics provides insights into genome evolution. However, most of the plant genomic sequences currently available have a long evolutionary timeframe which greatly reduces the power of the analyses. To better investigate the recent evolution of an important class of gene family, we have performed comparative genomics on species of the *Oryza* genus. It allowed us to look closer into the homologous regions at an increased resolution, which afforded us better capacity to understand mechanisms behind genomic rearrangements. High resolution WRKY evolution schemes are easier to achieve currently in the *Oryza* genus to the green-lineage specific nature of the proteins as well as availability of the rice genomic resources. These results provide valuable insights into the WRKY gene family in rice, a highly valuable food crop, and also help identify candidate genes that may confer favorable traits in the domestication of rice.

## Materials and methods

### Collection of genomic data sets

Complete amino acid and full-length complementary DNA (cDNA) sequences of *Oryza barthii*, *Oryza glaberrima*, *Oryza brachyantha*, *Oryza glumaepatula*, *Oryza meridionalis*, *Oryza nivara*, *Oryza punctata* and *Oryza rufipogon* were downloaded from Ensembl Plants (http://plants.ensembl.org/)*. Oryza sativa* subsp. *indica* genomes, R498 and MH63, were collected from two different *indica* varieties^41,42^. Using full-length amino acid sequences of WRKY proteins previously identified from *Oryza sativa* subsp. *japonica*^5,43^, a Hidden Markov model was constructed and used to identify WRKY proteins within each aforementioned *Oryza* species. HMMER 3.2b2, with a cut-off E-value of 0.001, was used to identify putative WRKY proteins. For genes with several isoforms the longest isoform was retained unless a shorter isoform contained a full WRKY domain. HMM identified WRKY proteins, retained isoforms, exceptions and discarded isoforms are documented in (Supplementary Tables S6 and S7).

### Orthologous assignment

After identifying WRKY proteins in each species, WRKYs were categorized into orthologous groups according to bidirectional BLASTp against *O. sativa* subsp. *japonica* (Supplementary Table S3). High scoring pairs in both directions were assigned as an ortholog to the matching WRKY protein in *O. sativa* subsp. *japonica.* WRKY proteins that could not be matched to an ortholog in *O. sativa* subsp. *japonica* were included in Supplementary Table S7. The convention in a previous review was used to name WRKY proteins^7^.

### Classification of WRKY genes

Full-length and WRKY domain sequences of WRKY proteins extracted using an in-house PERL program (Supplementary Table S8) were aligned using the MUSCLE program imbedded in MEGA7.0^44^ and classified according to groups solely on the basis of their WRKY and zinc-finger like conserved domains. Classification rules mentioned in previous publications were followed^1,5^. Briefly, WRKY proteins were sorted out into four main Groups—I, II, III and IV. Group I WRKY proteins contain two WRKY domains and two zinc finger motifs where the group can be further subdivided into Ia or Ib if it either has C_2_H_2_ zinc finger motif or C_2_HC, respectively. The C_2_ signature of the zinc fingers in both N-terminal and C-terminal WRKY domains are CX_4_C. Group II contains one WRKY domain with a C_2_H_2_ zinc finger motif and further subdivided into five subgroups based on the specific sequences found in its zinc finger. The C_2_ signature of the zinc finger in Subgroup IIa is **C**X_5_**C**PVKKK(L/V)Q, IIb CX_5_CPVRKQVQ, IIc CX_4_C, IId CX_5_CPARKHVE and IIe CX_5_CPARK(Q/M)V(E/D). Group III contains one WRKY domain and a C_2_HC zinc finger. WRKY proteins that either miss a WRKY or a zinc finger motif were relegated into Group IV^5^. It is important to note that WRKY genes are designated as Group IV when they contain no complete WRKY domains. This nomenclature is for the ease of presentation and based only on this indication of the total or partial loss of the WRKY domain and not phylogeny. In other words, unlike other groups, Group IV genes do not therefore cluster together on the phylogenetic tree.

### Visualization of chromosomal location of WRKY genes

Locus coordinates for WRKY genes and chromosome sizes of each genome were determined based on the GFF3 annotation data of the downloaded sequences (see Collection of Genomic Data Sets). MAPchart2.3^45^ was used to visually map out the WRKY genes.

### Structure and domain visualization

Motifs of identified WRKY proteins were analyzed using the MEME Suite 5.0.3 in the MEME website^46^. WRKY transcription displays were built using web-based tools developed in our lab^47^ and functional protein domains were identified using the ScanProsite tool^48^.

### Phylogenetic analysis

WRKY domain protein sequences extracted from each *Oryza* species were aligned using MUSCLE in MEGA7.0 with default settings. Group IVs were excluded due to the heavily degraded state of the WRKY domains. The alignments were used as inputs for RAxML within the Cipres portal to construct a phylogenetic tree^49^. RAxML was run under JTT protein matrix for 1,000 bootstraps. The RAxML output was visualized in iTOL^50^.

### Reconciliation tree

Gene duplication and loss events were predicted using NOTUNG v2.9^51^. Species tree of the different *Oryza* species used in the study was built from the NCBI Taxonomy Browser Gene. Maximum likelihood gene tree of representative *WRKY57* orthologs was built using RAxML from multiple sequence alignment of the full gene sequence with 1,000 bootstrap replicates and rearranged with NOTUNG v2.9 to reduce bias from weakly supported branches. Using the Duplication-Loss (DL) model, the rearranged maximum likelihood tree of representative WRKY orthologs were reconciled with the species tree. Standard parsimony weight parameters within the NOTUNG v2.9 program were used, specifically, 1.5 for duplication and 1.0 for loss.

### Synteny

Gene synteny was visualized using the Circos program^52^. Paralogs between chromosomes were identified using OrthNet ID from the CLfinder-OrthNet pipeline^53^. LOC_Os12g02440.4, LOC_Os12g40570.2, MH04t0557400-02, MH11t0647900-02, ORUFI12G19820.2, and OsR498G0409013800.01.P02 are exceptions to the longer isoform filtering, because they were more complete WRKY proteins or were more comparable to their orthologues. Protocols provided by the CLfinder-Orthnet pipeline were followed. Initial inputs for the pipeline were screened such that the loci with the longest protein isoforms were retained. One-to-one bidirectional BLASTN was used to collect High Scoring Pairs (HSP), which were filtered out if they had < 40% coverage relative to the query sequence. CLfinder-Orthnet clustering were produced with default settings. Paralogs were determined based on identical OrthNet, supplemented by inspection of the aligned full protein sequences. Supercomputer clusters on our campus was utilized to execute the pipeline.

### Co-linearity, transposition, and duplication

Data about co-linearity, transposition, and duplication was recovered via CLfinder-Orthnet pipeline^53^.

### Calculation of selection pressure and identification of positive selection

To detect positive selection that has occurred in some specific sites in the WRKY proteins, codon-based likelihood methods were run using the CODEML package in PAML ver 4.9. Full protein sequences of single copy orthologs were filtered for confidence using the GUIDANCE2 server^54^ with default cutoff value of 0.93, corresponding to 12% false positive rate and 78% true positive rate. Filtered sequences were backtranslated into coding domain sequences with EMBOSS Backtranseq^55^ using the codon usage table for *Oryza sativa.* PAL2NAL^56^ was used to convert protein multiple sequence alignments and corresponding backtranslated CDS into codon alignments using the Universal Codon Table and tree files used as input files for PAML.

Maximum likelihood estimates of the selection pressure were measured by nucleotide substitution rate (*dN/dS*) of non-synonymous (*dN*) to synonymous (*dS)*. Several models were formalized to test the process of evolution explicitly. In predicting which sites have been subject to selection, site model was used. This allows ω to vary at different sites in the gene. We used two pairwise likelihood ratio test: M1a vs M2a and M7 vs M8 to test for site specific codon evolution. The one-ratio model (M0) was used to obtain the average ω value for the genes. The Nearly Neutral model (M1a) includes two classes of sites (0 ≤ ω < 1 and ω = 1). The positive selection model (M2a) includes three classes of sites (0 ≤ ω < 1, ω = 1, and ω > 1). The β model (M7) uses the flexible β distribution to describe ω variation among sites and includes ten classes of sites with ω ≤ 1. The β and ω model (M8) uses the same distribution as M7 but includes 11 classes of sites on all lineages, 10 with ω ≤ 1, and 1 with ω > 1. The parameter estimates (ω ratios) and likelihood scores were calculated for two pairs of models and are detailed in Supplementary Table S9.

To test for branch specific dN/dS rates after duplication events, we used the reconciled gene trees to identify the branches immediately after the duplications and mark them as foreground branches. We did a branch model likelihood ratio test to compare the null model with a single rate across the whole tree and the alternative model with two rates, one for the foreground branches after duplication, and one for the rest of the background branches.

## Supplementary Information

Supplementary Figures.

Supplementary Table S1.

Supplementary Table S2.

Supplementary Table S3.

Supplementary Table S4.

Supplementary Table S5.

Supplementary Table S6.

Supplementary Table S7.

Supplementary Table S8.

Supplementary Table S9.

## Data Availability

The data underlying this article are available in its online supplementary material. The genomes of 11 species/subspecies were obtained from EnsemblPlants with the accession number GCA_001433935.1 for *Oryza sativa* subsp. *japonica*, GCA_000182155.2 for *Oryza barthii*, GCA_000231095.2 for *Oryza brachyantha*, GCA_000576495.1 for *Oryza glaberrima*, GCA_000576495.1 for *Oryza glumaepatula*, GCA_000338895.2 for *Oryza meridionalis*, GCA_000576065.1 for *Oryza nivara*, GCA_000573905.1 for *Oryza punctata*, GCA_002151415.1 for *Oryza sativa* subsp. *indica* cv. R498, and LNNK00000000 for *Oryza sativa* subsp. *indica* cv. MH63. All software used in this project are publicly available.
