# Supplementary material for: Dynamic differential evolution schemes of WRKY transcription factors in domesticated and wild rice
Source: Sci Rep. 2021 Jul 21;11:14887. doi: 10.1038/s41598-021-94109-4 (PMC8295372; doi:10.1038/s41598-021-94109-4)
Supplement: Supplementary file 1 — Supplementary Figures. [file 41598_2021_94109_MOESM1_ESM.pdf]

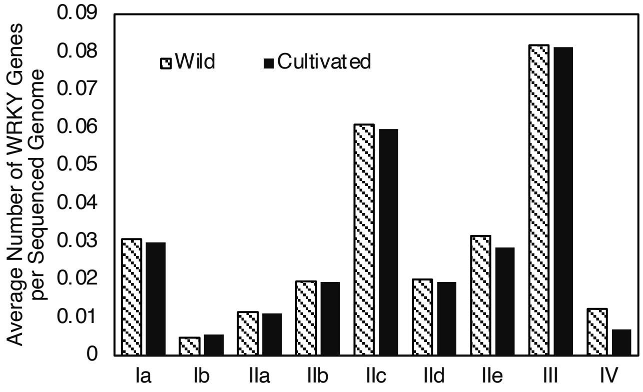

Supplementary Figure S1. Distribution of WRKY genes within wild species and domesticated species. Cultivated species include Asian (*Oryza sativa)* and African (*Oryza glaberrima*) cultivated rice.

Dynamic differential evolution schemes of WRKY transcription factors in domesticated and wild rice

Anne J. Villacastin, Keeley S. Adams, Rin Boonjue, Paul J. Rushton, Mira Han and Jeffery Q. Shen

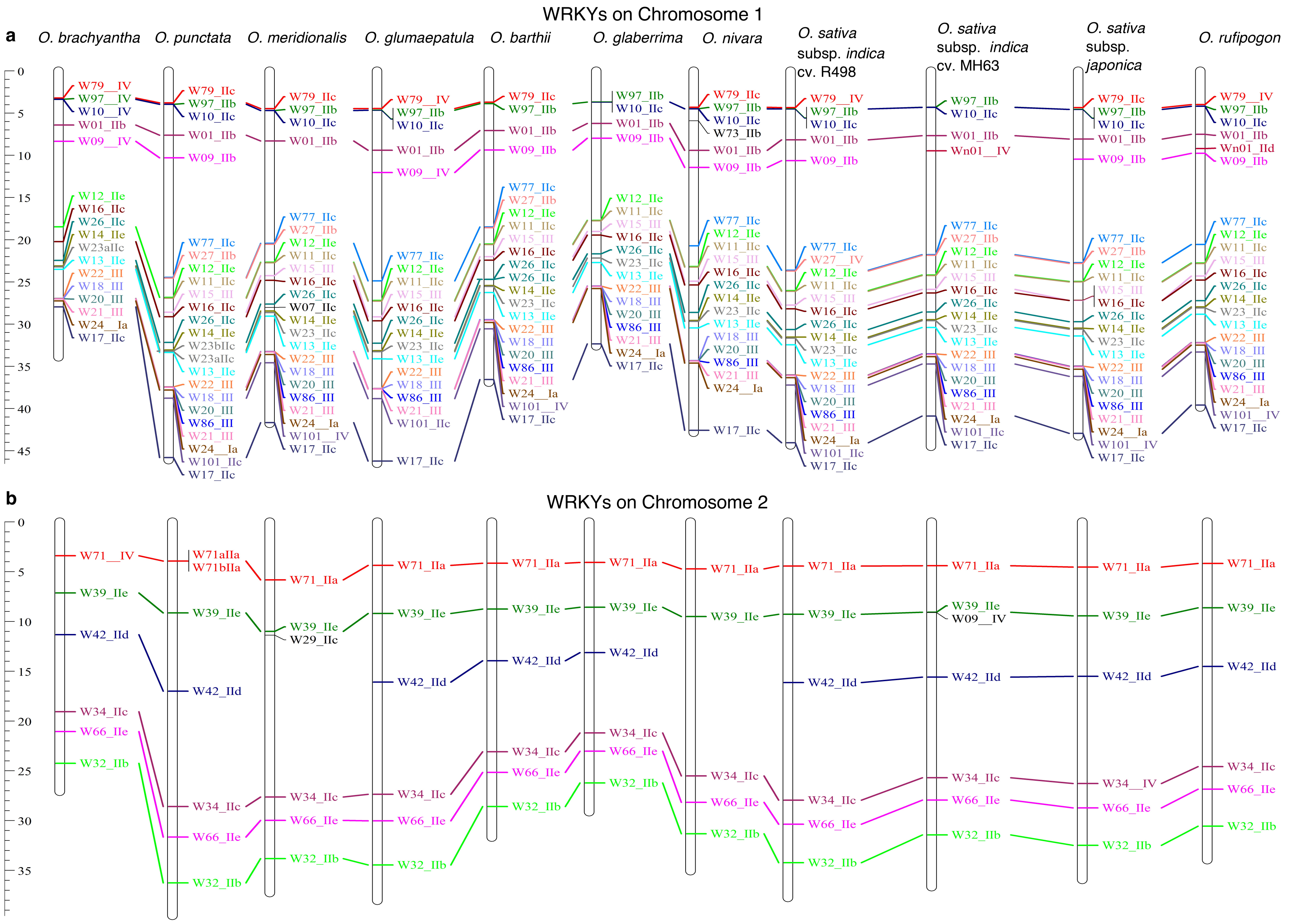

Supplementary Figure S2. Visualization of *WRKY* genes on *Oryza* species chromosomes 1-12. Vertical columns represent chromosomes scaled to length in mega base pairs (Mb). Species are arranged based on earliest species divergence to latest from left to right. The color codes represent orthology across *Oryza* species.

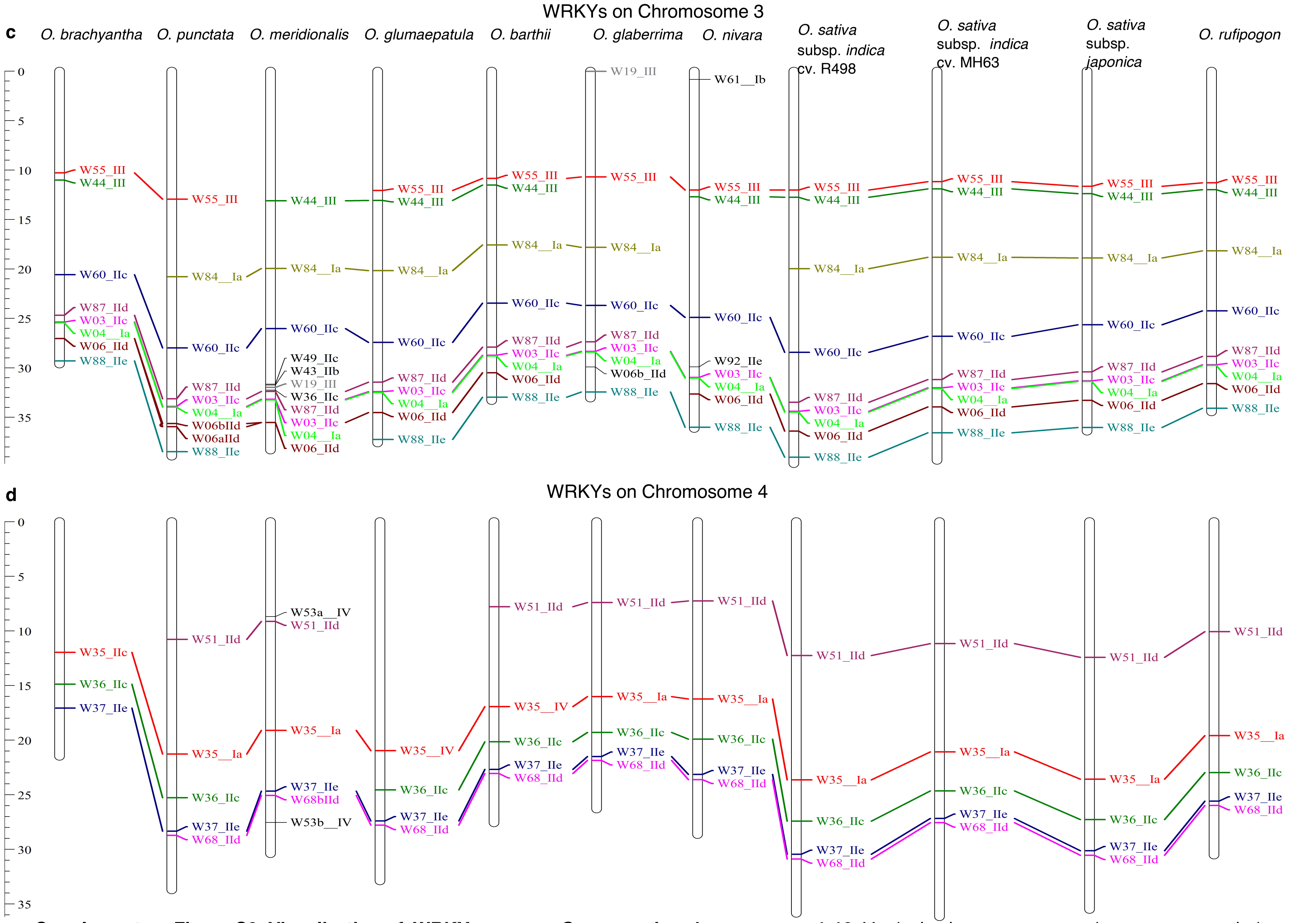

Supplementary Figure S2. Visualization of *WRKY* genes on *Oryza* species chromosomes 1-12. Vertical columns represent chromosomes scaled to length in mega base pairs (Mb). Species are arranged based on earliest species divergence to latest from left to right. The color codes represent orthology across *Oryza* species.

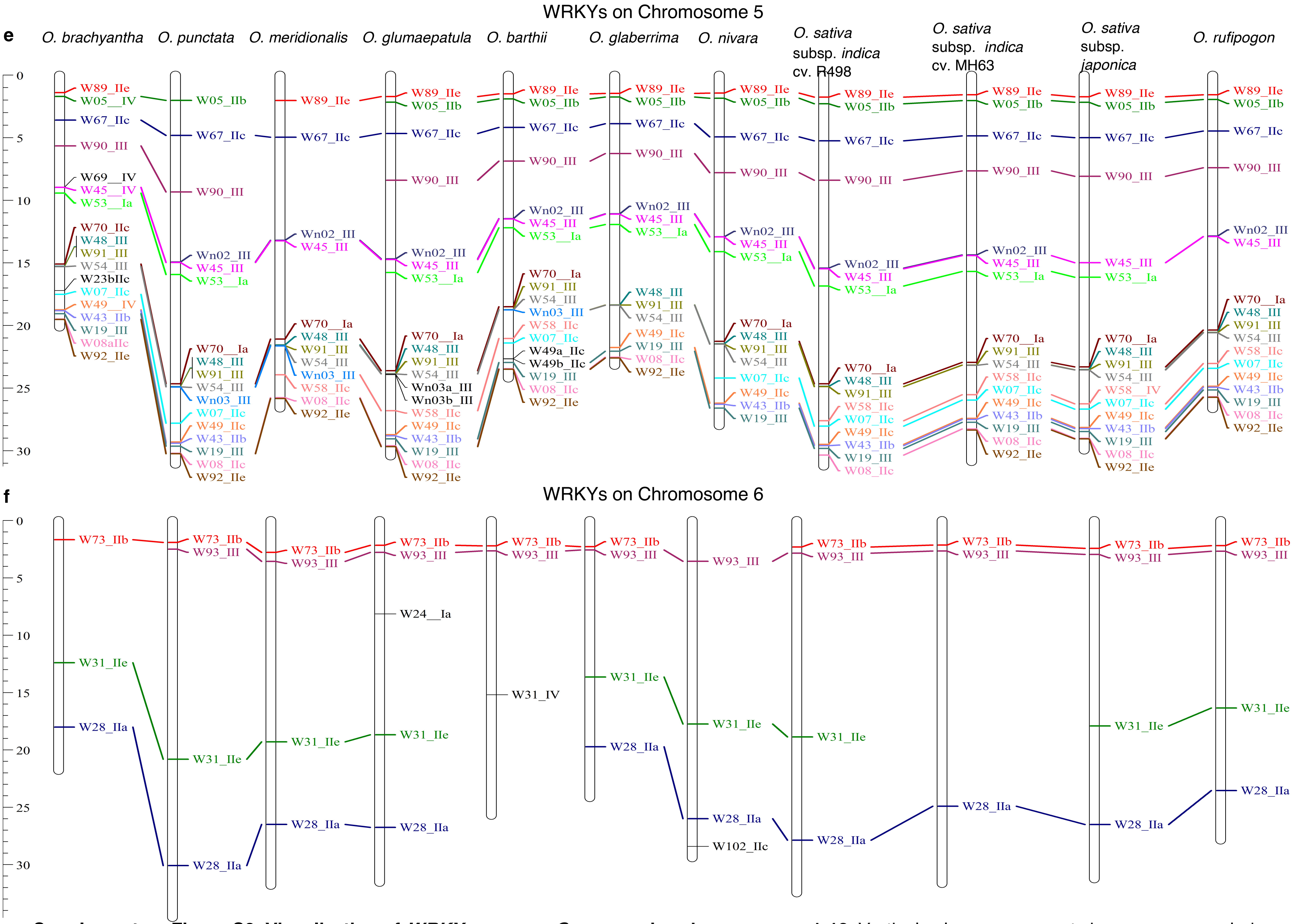

Supplementary Figure S2. Visualization of *WRKY* genes on *Oryza* species chromosomes 1-12. Vertical columns represent chromosomes scaled to length in mega base pairs (Mb). Species are arranged based on earliest species divergence to latest from left to right. The color codes represent orthology across *Oryza* species.

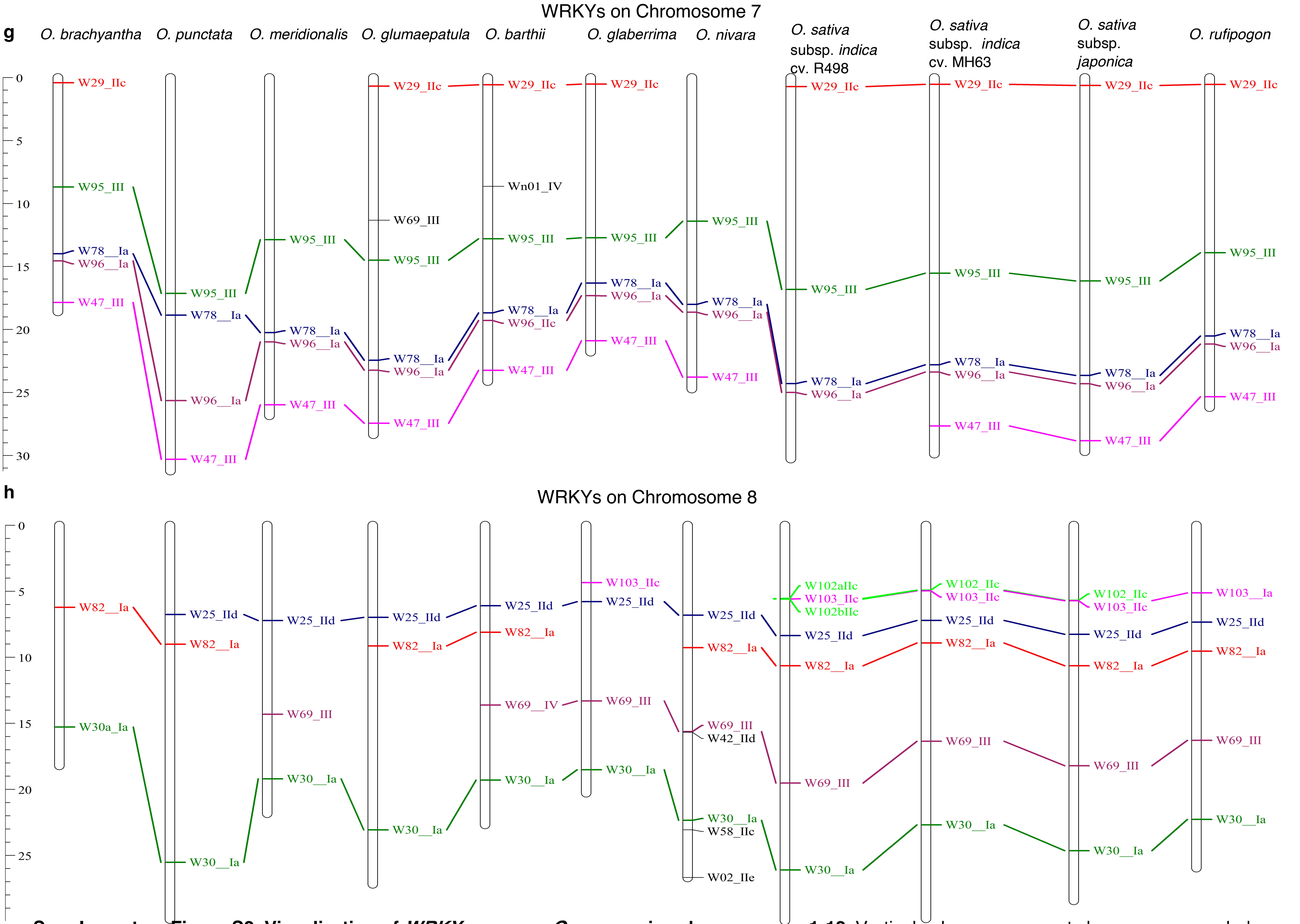

Supplementary Figure S2. Visualization of *WRKY* genes on *Oryza* species chromosomes 1-12. Vertical columns represent chromosomes scaled to length in mega base pairs (Mb). Species are arranged based on earliest species divergence to latest from left to right. The color codes represent orthology across *Oryza* species.

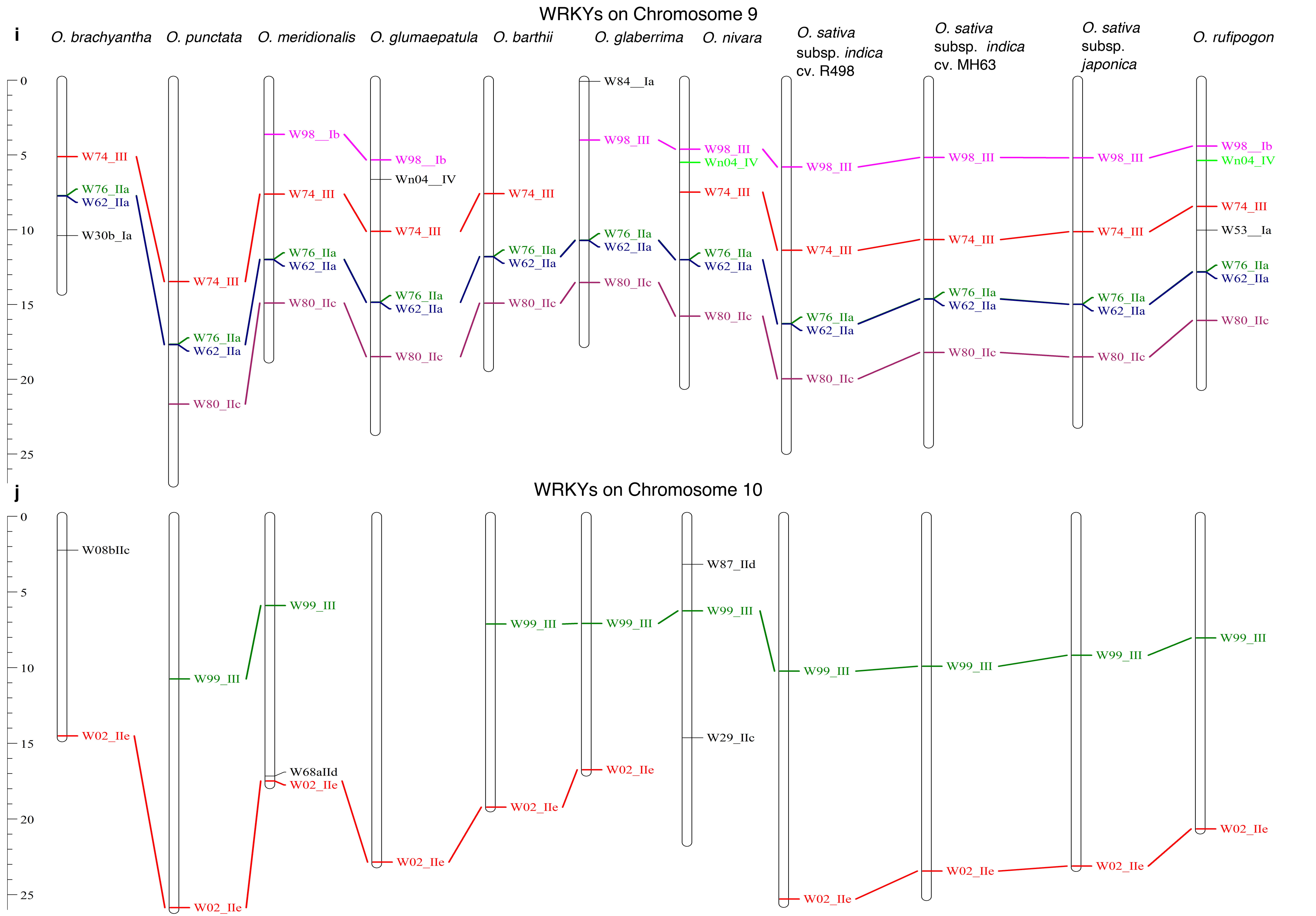

Supplementary Figure S2. Visualization of *WRKY* genes on *Oryza* species chromosomes 1-12. Vertical columns represent chromosomes scaled to length in mega base pairs (Mb). Species are arranged based on earliest species divergence to latest from left to right. The color codes represent orthology across *Oryza* species.

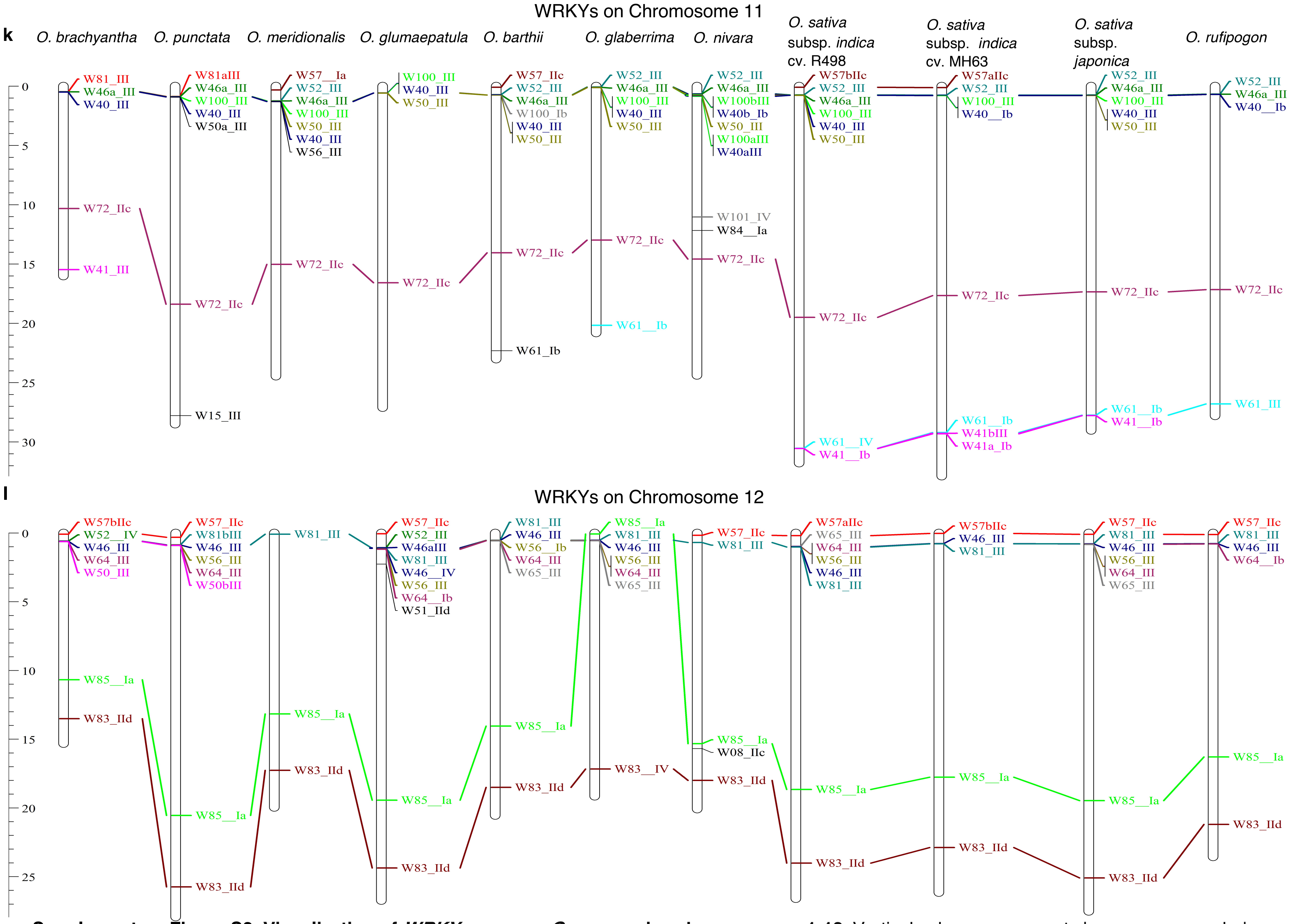

Supplementary Figure S2. Visualization of *WRKY* genes on *Oryza* species chromosomes 1-12. Vertical columns represent chromosomes scaled to length in mega base pairs (Mb). Species are arranged based on earliest species divergence to latest from left to right. The color codes represent orthology across *Oryza* species.

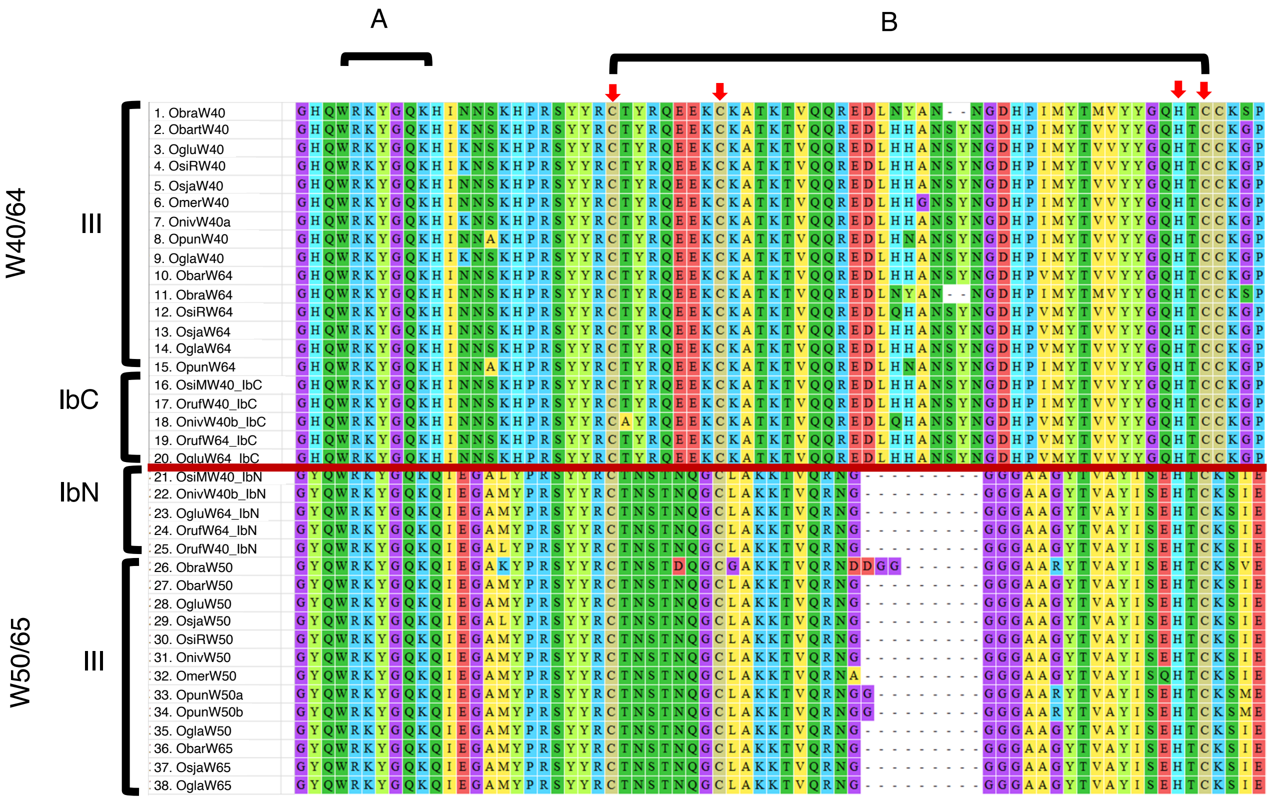

Supplementary Figure S3. Protein alignment of WRKY40, WRKY50, WRKY64, and WRKY65. The N-terminal and C-terminal WRKY domains of Group Ib WRKYs are visibly divided into two sets of sequences: WRKY40/64-like and WRKY50/65-like. Label A indicates WRKYGQK motif. Label B indicates Zinc-finger motif; C_2HC are marked by red arrows.

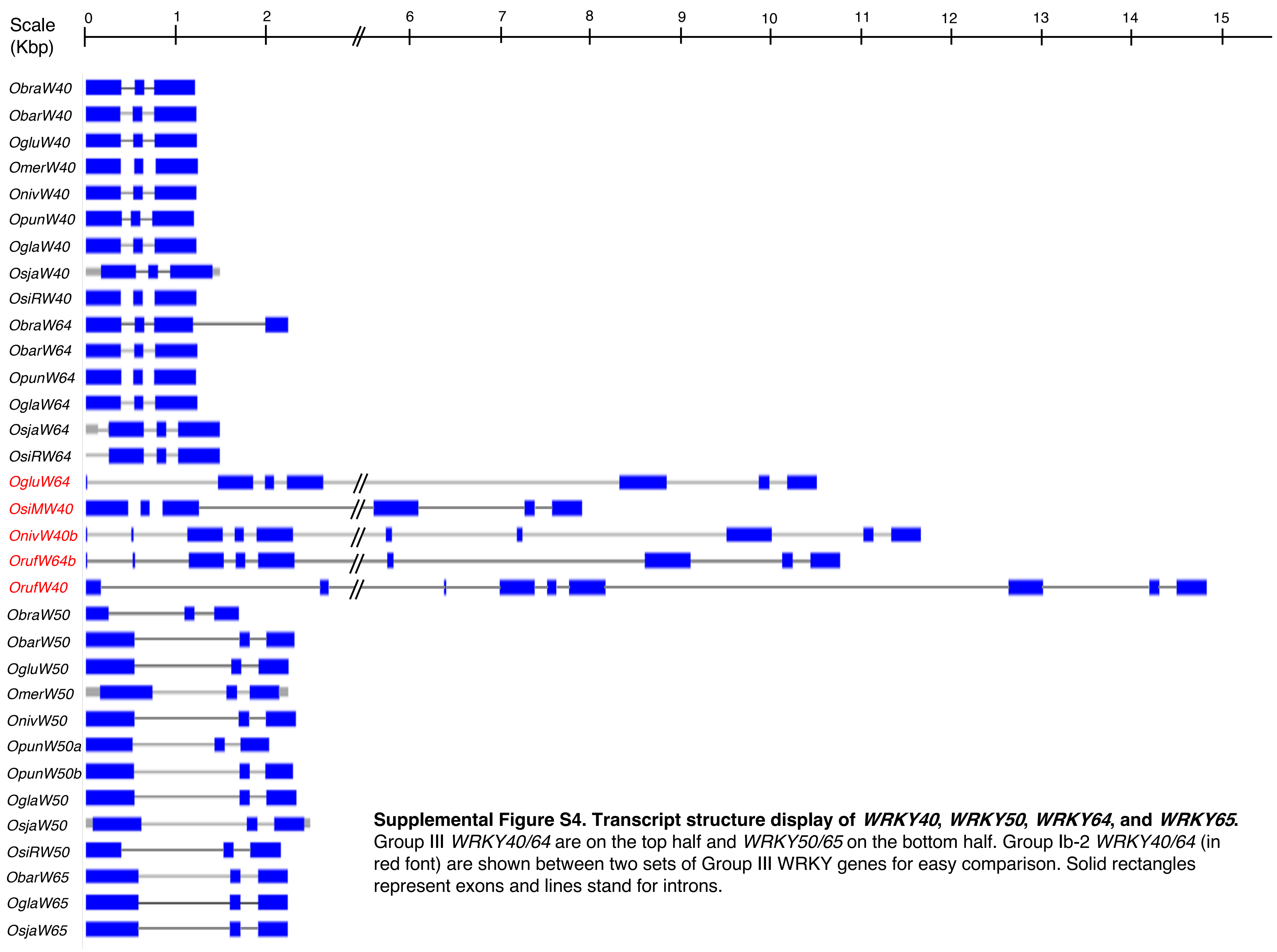

Supplemental Figure S4. Transcript structure display of *WRKY40*, *WRKY50*, *WRKY64*, and *WRKY65*. Group III *WRKY40/64* are on the top half and *WRKY50/65* on the bottom half. Group Ib-2 *WRKY40/64* (in red font) are shown between two sets of Group III WRKY genes for easy comparison. Solid rectangles represent exons and lines stand for introns.

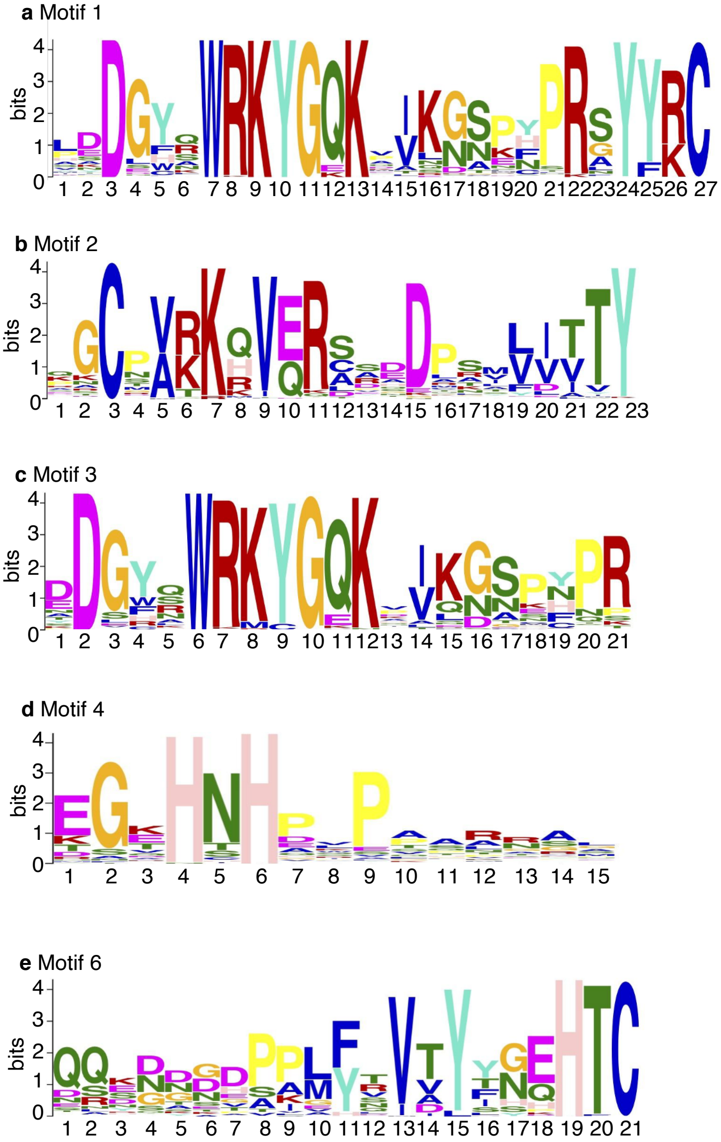

Supplementary Figure S5. Sequences of top motifs analyzed in 979 WRKY proteins excluding Group IV subgroups. a. Motif predicted in 1000 sites **b.** Motif predicted in 766 sites ***c.*** Motif predicted in 108 sites **d.** Motif predicted in 108 sites *e.* Motif predicted in 218 sites

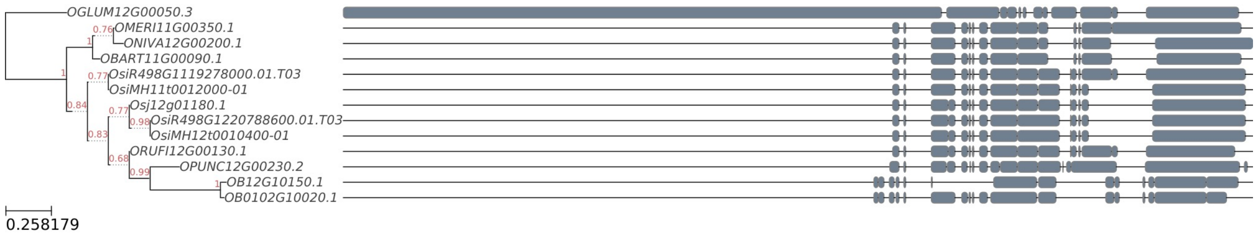

Supplementary Figure S6. Phylogenetic tree of WRKY57 subfamily. Maximum likelihood gene tree was built using RAxML from multiple sequence alignment of full gene sequence with 1,000 bootstrap replicates. Schematic representation of the multiple sequence alignment is shown on the right panel.

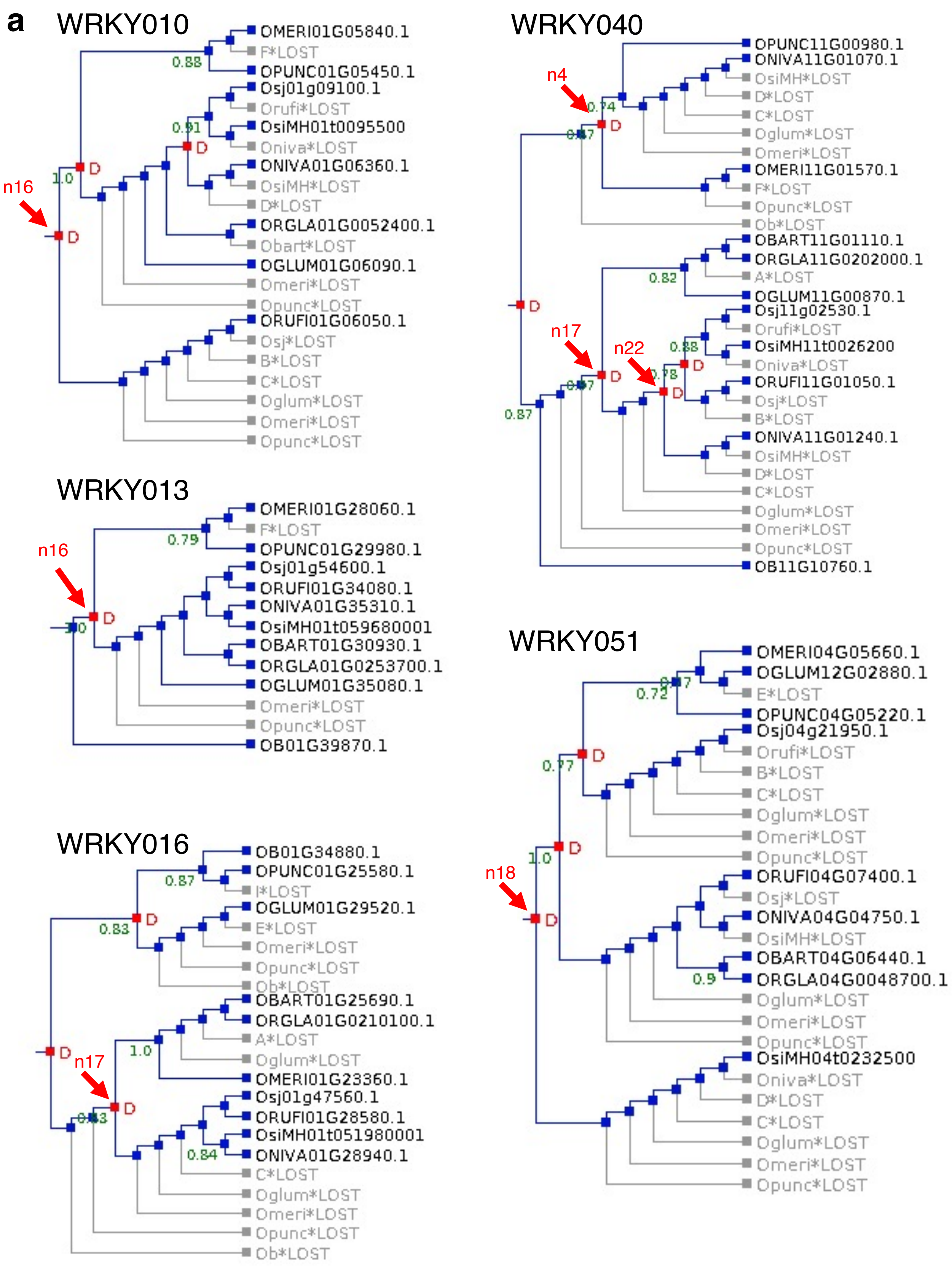

Supplementary Figure S7. Notung reconciled trees of WRKY subfamilies with significantly different rate of evolution after duplication event. Red arrows indicate duplication nodes analyzed for rate evolution using PAML branch model (Supplementary Table S9)

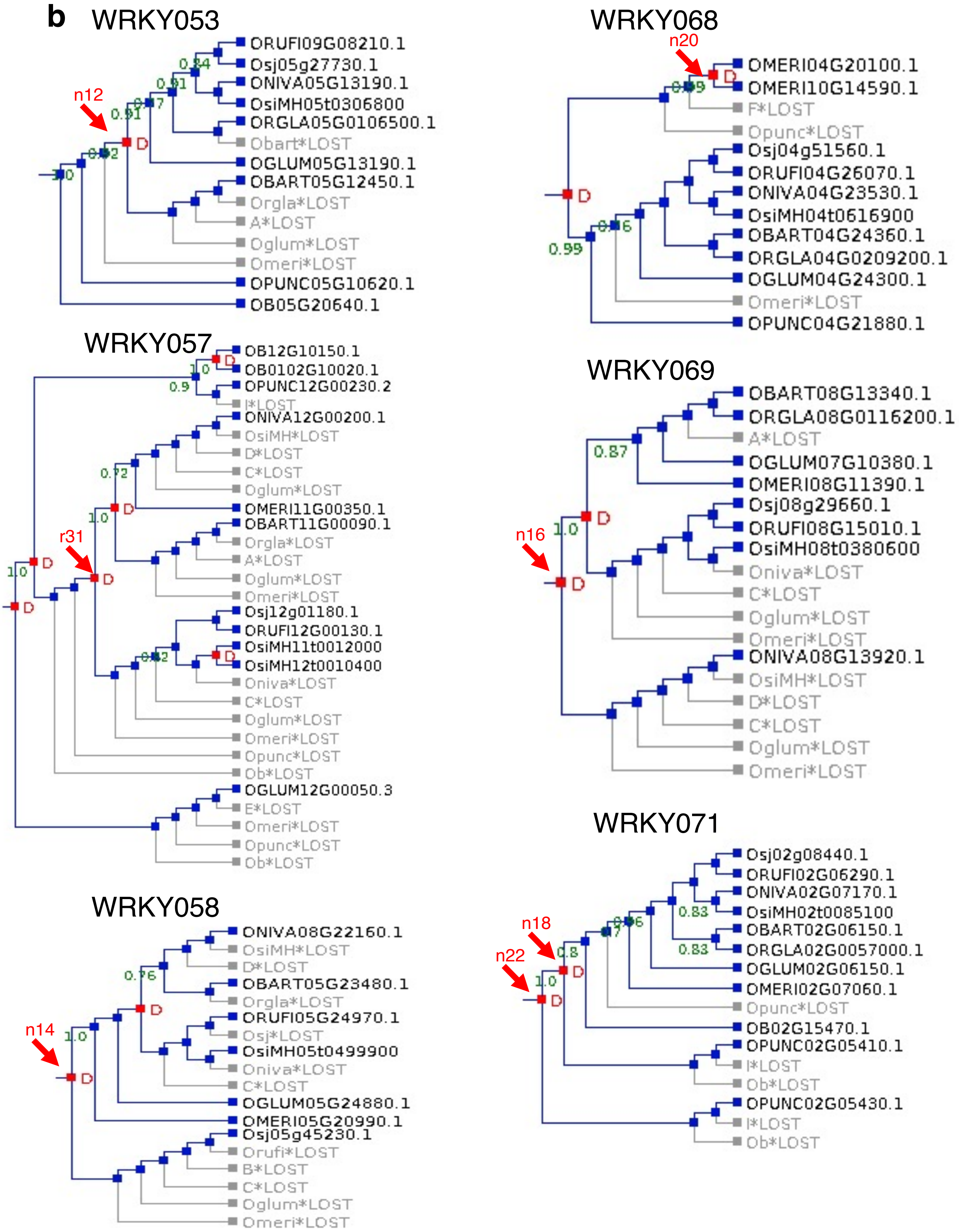

Supplementary Figure S7. Notung reconciled trees of WRKY subfamilies with significantly different rate of evolution after duplication event. Red arrows indicate duplication nodes analyzed for rate evolution using PAML branch model (Supplementary Table S9)

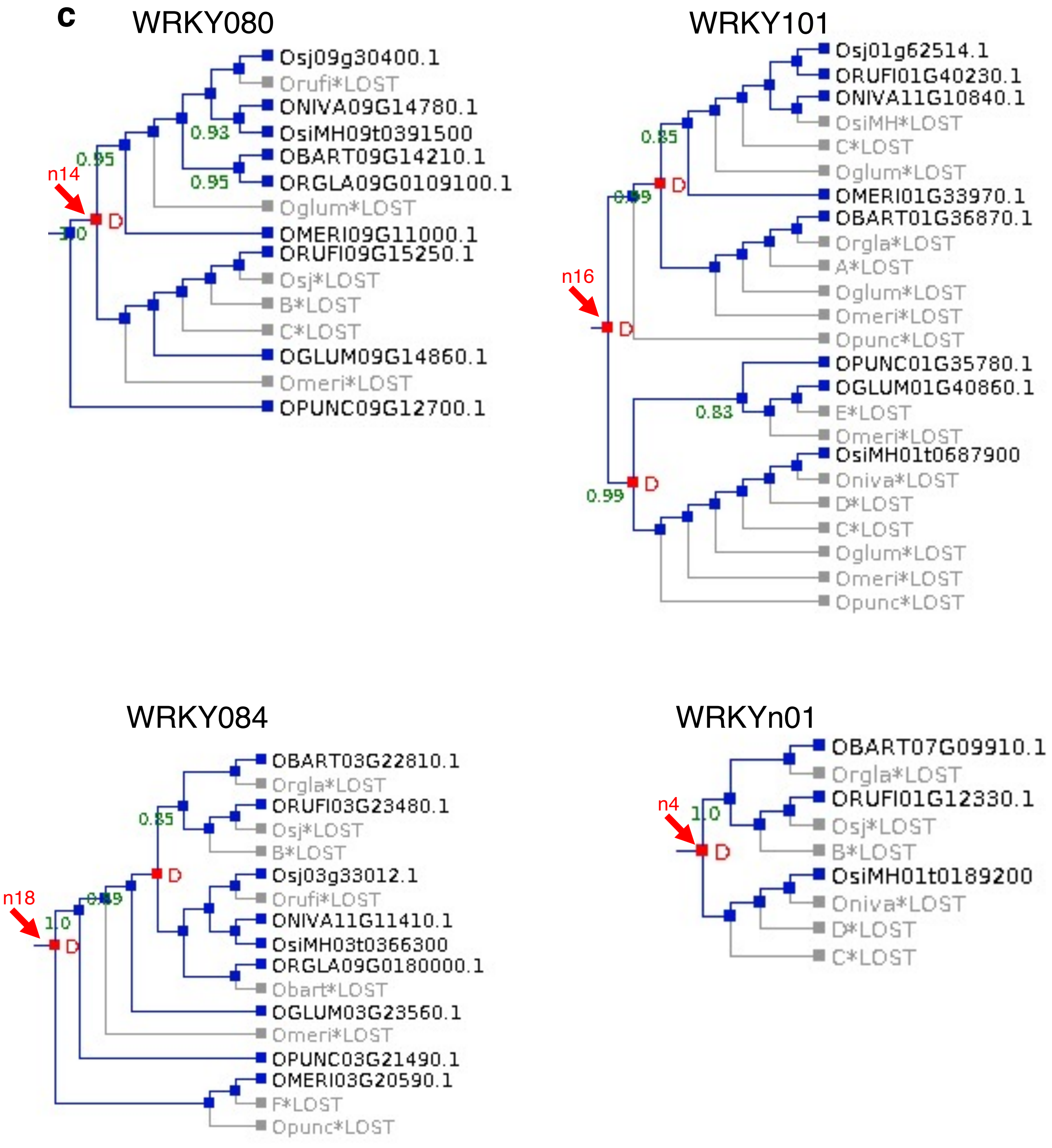

Supplementary Figure S7. Notung reconciled trees of WRKY subfamilies with significantly different rate of evolution after duplication event. Red arrows indicate duplication nodes analyzed for rate evolution using PAML branch model (Supplementary Table S9)
